# The effect of substrate wettability and modulus on gecko and gecko-inspired synthetic adhesion in variable temperature and humidity

**DOI:** 10.1038/s41598-020-76484-6

**Published:** 2020-11-12

**Authors:** Christopher T. Mitchell, Cem Balda Dayan, Dirk-M. Drotlef, Metin Sitti, Alyssa Y. Stark

**Affiliations:** 1 Department of Biology, Villanova University, 800 E. Lancaster Ave., Villanova, PA 19085 USA; 2 Physical Intelligence Department, Max Planck Institute for Intelligent Systems, 70569 Stuttgart, Germany

**Keywords:** Ecophysiology, Biomechanics, Bioinspired materials

## Abstract

Gecko adhesive performance increases as relative humidity increases. Two primary mechanisms can explain this result: capillary adhesion and increased contact area via material softening. Both hypotheses consider variable relative humidity, but neither fully explains the interactive effects of temperature and relative humidity on live gecko adhesion. In this study, we used live tokay geckos (*Gekko gecko*) and a gecko-inspired synthetic adhesive to investigate the roles of capillary adhesion and material softening on gecko adhesive performance. The results of our study suggest that both capillary adhesion and material softening contribute to overall gecko adhesion, but the relative contribution of each depends on the environmental context. Specifically, capillary adhesion dominates on hydrophilic substrates, and material softening dominates on hydrophobic substrates. At low temperature (12 °C), both capillary adhesion and material softening likely produce high adhesion across a range of relative humidity values. At high temperature (32 °C), material softening plays a dominant role in adhesive performance at an intermediate relative humidity (i.e., 70% RH).

## Introduction

The gecko adhesive system has sparked intense research and innovation for more than two decades^1–15^. However, despite significant interest in the morphology, evolutionary history, and biomechanical principles of the gecko adhesive system, there is still uncertainty about the governing mechanisms of gecko adhesion. Specifically, the potential roles of capillary adhesion and material softening on gecko adhesive performance in humid environments have often been debated^16–20^. Habitat diversity of geckos suggests that geckos must maintain adhesion in a variety of contexts, including hot and humid tropical environments^21–25^, thus understanding the adhesive mechanism in these conditions may be key to understanding diversification of the gecko adhesive system.

Geckos use microscopic, hair-like structures (setae) to amplify attractive van der Waals forces of the superhydrophobic adhesive toe pads^1,26–32^. Although a van der Waals-based adhesive system creates a robust and reversible adhesive force, common environmental factors may disrupt the functionality of the system. For example, thin water layers have the potential to reduce van der Waals forces to zero when separating setae from a substrate by as a little as 20 nm^33^. Despite this, gecko adhesion increases as thin water layer thickness from ambient relative humidity (RH) increases^16–19,34^. Two hypotheses have been proposed to explain this result: capillary adhesion and material softening. Gecko adhesion increases as the substrate becomes more hydrophilic, supporting the hypothesis that capillary bridges between gecko setae and the water-attracting substrate enhance adhesion^16–18^. Likewise, at high humidity (> 70% RH) the setal material (primarily keratin associated proteins and lipids^35–38^) softens, supporting the hypothesis that soft setae increase the interfacial contact area and subsequently increase van der Waals forces^19,20^. The results of these studies are difficult to reconcile^14,34,39^, and none consider the possibility that capillary adhesion and material softening are not mutually exclusive.

In addition to fluctuations in RH, other environmental factors may also alter gecko adhesive performance. For instance, ectothermic geckos may be particularly susceptible to variation in temperature due to potential limitations on muscle and kinematic performance^40–42^. However, body temperature independently has no effect on live gecko adhesion^43,44^. Instead, gecko adhesive performance is strongly related to ambient temperature when tested across a range of RH^34,39^. Specifically, at low temperature (12 °C), gecko adhesion increases with increasing RH^34^, supporting results from separated setae tested at room temperature^16,19^. However, at high temperature (32 °C), RH has no effect on live gecko adhesion^34^. A nanoscopic length scale model attempted to explain the temperature dependency of this relationship, but ultimately the coupled effects of RH and temperature on gecko adhesion are still undetermined^39^.

Live gecko adhesion results and setal adhesion models in variable temperature and RH do not fully support either of the current hypotheses that explain gecko adhesion in variable RH (i.e., capillary adhesion, material softening). The purpose of this study is to decouple these hypotheses by testing the adhesive performance of live geckos and a gecko-inspired synthetic adhesive (GSA) model in variable temperature and RH conditions, and to consider that these hypotheses may not be mutually exclusive. To test our hypothesis, we used live geckos and GSAs because live gecko setal surface chemistry and modulus change in wet environments^20,35,45^, and GSA surface chemistry and modulus remains relatively constant. Thus, the qualitative comparison between live gecko and GSA adhesive performance in the same conditions allows us to explore the influence of capillary adhesion and material softening with and without confounding factors innate to the live animal system. We hypothesize that both capillary adhesion and material softening play a role in live gecko and GSA performance (i.e., are not mutually exclusive), and that these roles change dominance depending on environmental conditions (i.e., substrate wettability, adhesive material modulus, temperature, RH).

To test for an effect of capillary adhesion on gecko adhesive performance, we measured shear adhesive performance of live geckos and GSAs in variable temperature and RH conditions on hydrophobic [octadecyltrichlorosilane-self assembled monolayer (OTS-SAM) coated glass] and hydrophilic (untreated glass) substrates, which limit or support capillary adhesion, respectively. To explore the role of setal stiffness on gecko adhesive performance in variable temperature and RH conditions, we replicated our live gecko and GSA experiments with two additional GSAs that were either soft or stiff. Here, we compared adhesive performance of the model GSAs with artificially defined stalk modulus (soft, medium, stiff) across varying conditions (i.e., temperature, RH, substrate wettability). The results of this study improve our understanding of the gecko adhesive mechanism and gecko ecology related to the interaction of adhesive performance and relevant abiotic environmental conditions like temperature, RH, and substrate wettability.

## Results

Shear adhesion of live geckos and GSAs differed in response to temperature, RH, substrate wettability, and modulus (GSAs only). Of all possible interactions for each system (live gecko, GSA), only four two-way statistical interactions were significant. These are discussed below. All statistical results [i.e., F values (F), degrees of freedom (subscript numerical values), *p* value (*p*); Tables S1 and S2] and model considerations (i.e., tests for homogeneity of variance; Tables S3 and S4) are reported below and in the Supplementary Material. Means of the treatment groups were deemed different from one another only when the *p* value calculated from the statistical model was *p* ≤ 0.05.

### Effect of relative humidity and substrate wettability

**Figure 1:**
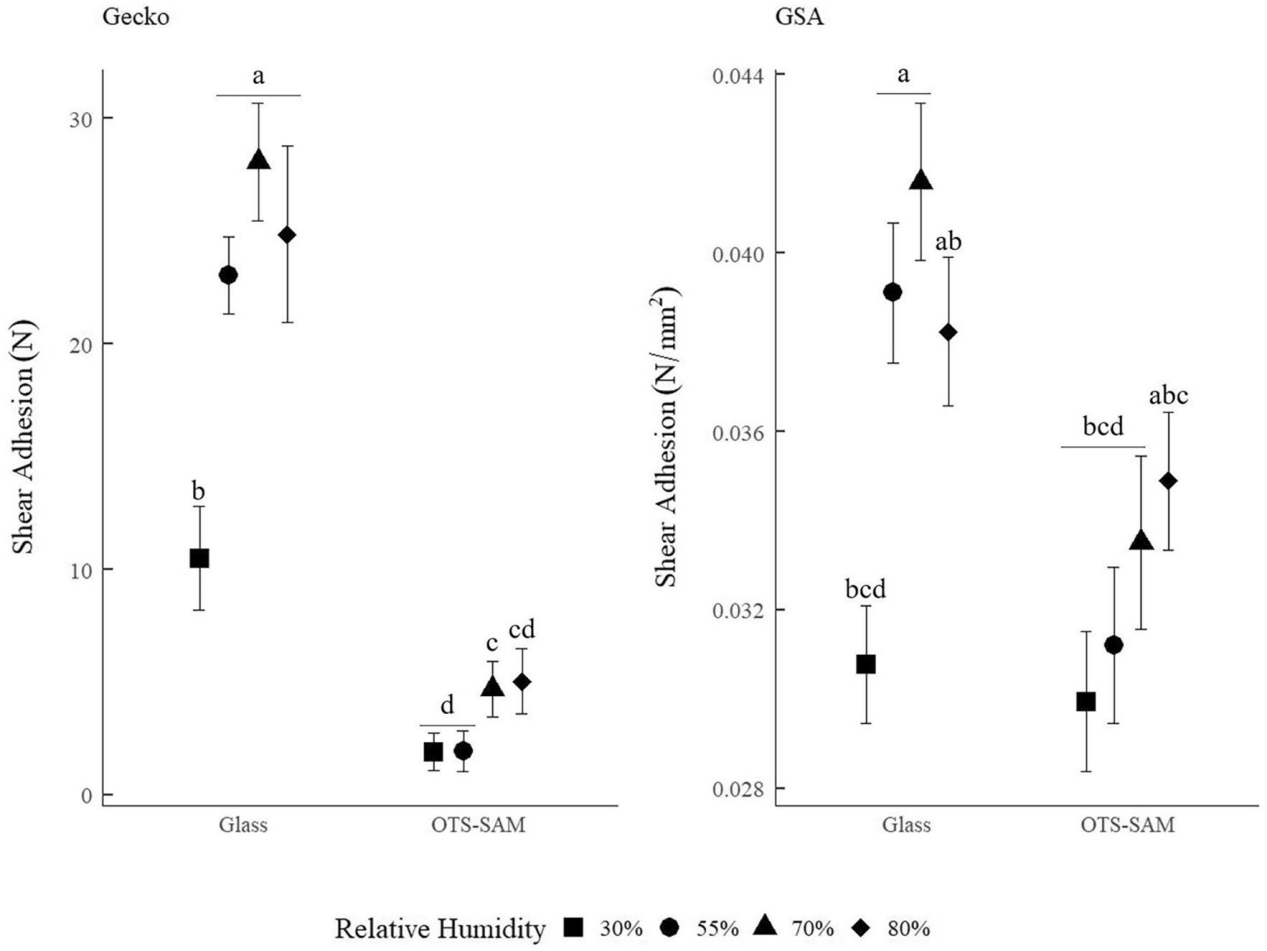
Tokay gecko (*Gekko gecko*; sample size = 7; left panel) and gecko-inspired synthetic adhesive (GSA; sample size = 5; right panel) shear adhesion (mean ± s.e.m.) in variable relative humidity (30%, 55%, 70%, and 80%) on hydrophilic (ca. 50° water contact angle) glass and hydrophobic (ca. 100° water contact angle) octadecyltrichlorosilane self-assembled monolayer (OTS-SAM) coated glass. Shear adhesion of live geckos is measured as maximum shear force resisted before sliding (N) and shear adhesion of GSAs is measured as maximum shear force resisted while sliding per unit area (N/mm^2^), matching previous work^34,47^. The means of treatment groups denoted with the same letter are not statistically different from one another according to Tukey post hoc pairwise statistical tests (see Tables S1 and S2 for a detailed explanation of statistical analysis and the model output).

Gecko and GSA shear adhesion differed in response to the interaction between RH and substrate wettability (F_3,90_ = 5.096, *p* = 0.0026; F_3,180_ = 5.638, *p* = 0.0010, respectively; Tables S1 and S2). On the hydrophilic untreated glass, live gecko and GSA shear adhesion increased between 30 and 55% RH. Gecko and GSA shear adhesion remained constant on glass at > 55% RH (Fig. 1). On the hydrophobic OTS-SAM coated glass, live gecko shear adhesion increased at ≥ 70% RH, while GSA adhesion was unaffected by RH (Fig. 1).

### Effect of relative humidity and temperature

**Figure 2:**
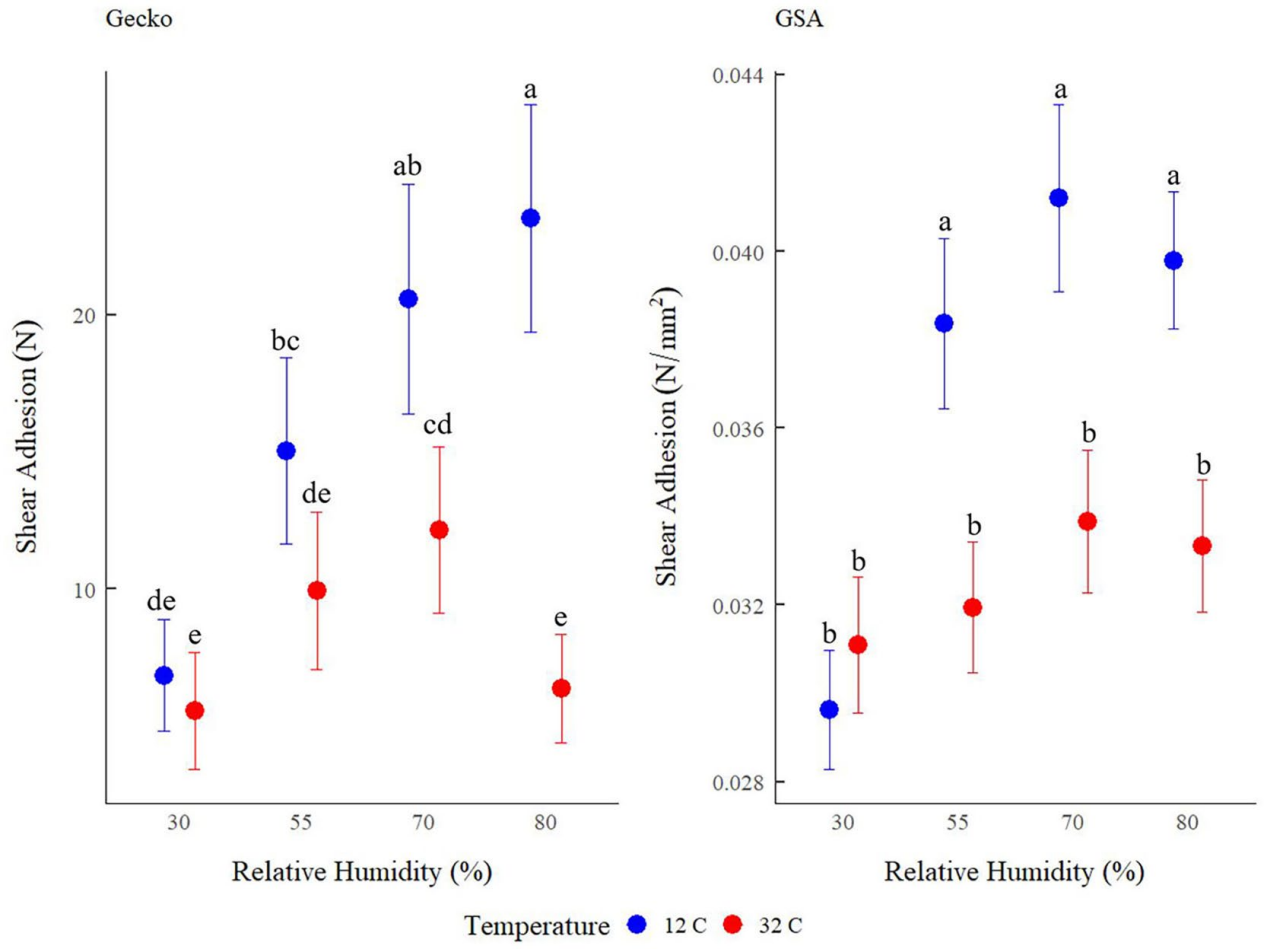
Tokay gecko (*Gekko gecko*; sample size = 7; left panel) and gecko-inspired synthetic adhesive (GSA; sample size = 5; right panel) shear adhesion (mean ± s.e.m.) in variable relative humidity (30%, 55%, 70%, and 80%) and temperature (12 °C and 32 °C). Shear adhesion of live geckos is measured as maximum shear force resisted before sliding (N) and shear adhesion of GSAs is measured as maximum shear force resisted while sliding per unit area (N/mm^2^), matching previous work^34,47^. The means of treatment groups denoted with the same letter are not statistically different from one another according to Tukey post hoc pairwise statistical tests (see Tables S1 and S2 for a detailed explanation of statistical analysis and the model output).

Gecko and GSA shear adhesion differed in response to the interaction between RH and temperature (F_3,90_ = 10.190, *p* < 0.0001; F_3,180_ = 5.828, *p* = 0.0008, respectively; Tables S1 and S2). In general, at low temperature (12 °C), gecko shear adhesion increased as RH increased. Similarly, GSA shear adhesion increased as RH increased, except that the increase occurred between 30 and 55% RH, and shear adhesion of all subsequent set points did not differ (Fig. 2). At high temperature (32 °C), gecko shear adhesion showed a slight peak at 70% RH, while GSA adhesion was unaffected by RH at 32 °C (Fig. 2).

### Effect of modulus and substrate wettability

**Figure 3:**
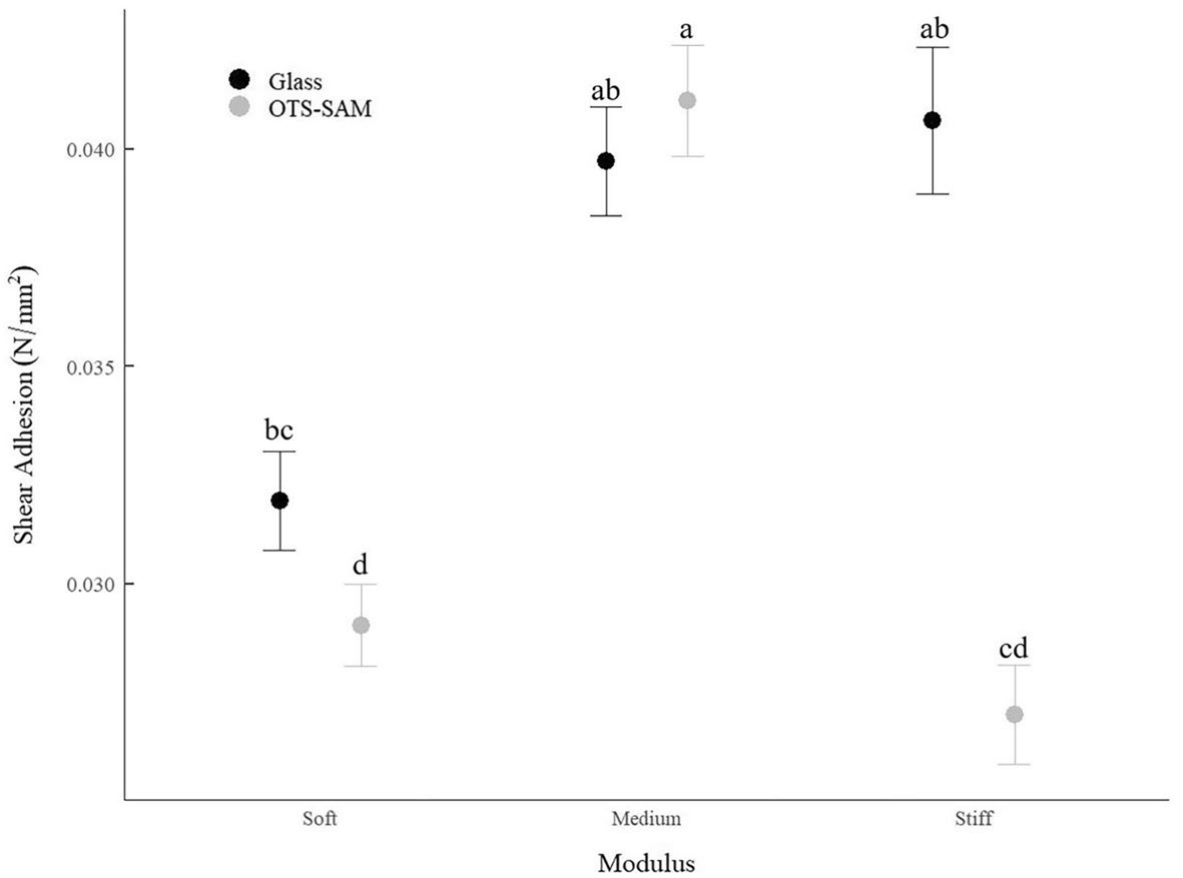
Shear adhesion (mean ± s.e.m.) of three gecko-inspired synthetic adhesives (GSAs; sample size = 5) with different modulus values (soft = 0.093 ± 0.0047 (s.d.) MPa; medium = 0.83 ± 0.020 (s.d.) MPa; stiff = 1.91 ± 0.140 (s.d.) MPa) on hydrophilic (ca. 50° water contact angle) glass and hydrophobic (ca. 100° water contact angle) octadecyltrichlorosilane self-assembled monolayer (OTS-SAM) coated glass. The means of treatment groups denoted with the same letter are not statistically different from one another according to Tukey post hoc pairwise statistical tests (see Table S2 for a detailed explanation of statistical analysis and the model output).

Shear adhesion of GSAs with artificially defined stalk modulus (soft, medium, stiff) differed in response to the interaction between GSA pillar stiffness and substrate wettability (F_2,180_ = 24.610, *p* < 0.0001; Table S2). On the hydrophilic glass, modulus had no effect on GSA shear adhesion. However, on the hydrophobic OTS-SAM coated glass, shear adhesion of the medium modulus GSA was higher than the stiff and soft modulus GSAs (Fig. 3).

### Effect of temperature and substrate wettability

Shear adhesion of live geckos differed in response to the interaction between temperature and substrate wettability (F_1,90_ = 32.040, *p* < 0.0001; Table S1). On the hydrophilic glass, gecko shear adhesion at 12 °C was 60% higher than shear adhesion at 32 °C. On the hydrophobic OTS-SAM coated glass, gecko shear adhesion at 12 °C was only 12% higher than shear adhesion at 32 °C (Fig. S1). Overall, gecko shear adhesion on the OTS-SAM coated glass was lower than shear adhesion on untreated glass.

## Discussion

Geckos are extremely diverse (i.e., > 1800 species^13^) and live in a multitude of habitats with a wide variety of environmental conditions (e.g., hot, humid, cool, dry). Despite significant interest in geckos, it is unclear how the gecko adhesive system simultaneously manages variation in ambient temperature and RH. Specifically, experimental results and computational models of single seta and setal array adhesion in variable RH suggest that either capillary adhesion or material softening explain gecko adhesive performance^16–20^. However, neither of these hypotheses fully explain gecko adhesion in variable RH and temperature, and they are not necessarily mutually exclusive^34,39^. Indeed, the results of this study suggest that in variable RH and temperature, both capillary adhesion and material softening influence gecko adhesion. Our results highlight several key interactions among temperature, RH, substrate wettability, and modulus in live gecko and GSA systems. These interactions are discussed below.

### Effect of relative humidity and substrate wettability

On hydrophilic glass, gecko adhesion and GSA adhesion are consistent and match adhesion behavior of other polymer GSAs in variable RH^46–48^. Using a GSA as a control for material softening and surface chemistry (i.e., GSAs experience little relative change in modulus and surface chemistry as function of water^49^), it is clear both systems are significantly influenced by capillary adhesion on hydrophilic glass (i.e., adhesion is higher at 70% RH than at 30% RH), similar to studies testing single setae and setal tips (spatula)^16,17^. On hydrophobic OTS-SAM coated glass, where we expect RH-induced water layers and thus capillary adhesion to be limited, GSA adhesion is unaffected by RH. Conversely, gecko adhesion generally increased at higher RH (70% and 80% RH; Fig. 1). This discontinuity highlights likely interactive effects of capillary adhesion and material softening in the gecko adhesive system. Specifically, the softening of gecko setae (material softening hypothesis) occurs on both hydrophilic and hydrophobic substrates at high RH (i.e., gecko setae soften at > 70% RH regardless of substrate^19,20^). However, the measurable increase in live gecko adhesion due to increased contact area from material softening (though changes in setal surface chemistry cannot be ruled out) at high RH appears to only be detectable when capillary adhesion is discouraged by substrate wettability (i.e., OTS-SAM coated glass).

### Effect of relative humidity and temperature

In general, at low temperature (12 °C), gecko and GSA adhesive performance increases as RH increases^34,47^. Because the GSA is not significantly affected by changes in modulus or surface chemistry, capillary adhesion appears to drive this response in low temperature. However, at high temperature (32 °C), gecko adhesion peaks at 70% RH, which was not detected previously^34^ and not observed in the GSA system (Fig. 2). Thus, the difference between gecko adhesive performance and GSA adhesive performance at high temperature likely signifies that either changes in setal surface chemistry or modulus are responsible for the observed enhancement of live gecko adhesion at high temperature and intermediately high RH (Fig. 2).

### Effect of modulus and substrate wettability

On hydrophilic glass, there is no statistical difference in GSA adhesive performance as a function of stalk modulus. However, the pairwise comparisons of adhesion on glass between soft and stiffer GSAs is nearly different in one instance (i.e., soft-medium GSA comparison *p* value is nearly *p* ≤ 0.05; soft-stiff: *p* = 0.1068; soft-medium: *p* = 0.0958). On hydrophobic OTS-SAM coated glass, shear adhesion of the medium GSA is higher than the soft and the stiff GSAs, suggesting that when capillary adhesion is ineffective (i.e., highly reduced water layers), stalk modulus significantly impacts adhesive performance and moderately soft stalks perform better. The difference in shear adhesive performance between the medium and stiff GSAs on hydrophobic OTS-SAM coated glass, but not hydrophilic glass, is significant given similarities between the GSA and gecko adhesive systems. Specifically, the modulus of gecko setae in humid conditions (i.e., 80% RH) is ca. 40% lower than the modulus of gecko setae in dry conditions^20^. In this experiment, we matched GSA modulus to live gecko modulus changes such that the modulus of the medium GSA is 40% lower than the modulus of the stiff GSA. Thus, the results of the GSA experiment support our observations in live geckos, and both systems are likely optimized for higher adhesion with moderately stiff stalks on hydrophobic but not hydrophilic substrates. This result highlights the importance of material properties on hydrophobic substrates that limit capillary adhesion.

On both hydrophobic and hydrophilic substrates, it is likely the slightly lower shear adhesion of the soft GSA is related to contact mechanics. Specifically, maximum shear adhesion of the stiff GSA is followed by a rapid decrease in tip contact, whereas maximum shear adhesion of the soft GSA is achieved incrementally as pillars buckle and slide. This suggests that very stiff stalks are “stiction” dominated (i.e. pillar adhesion drives overall shear adhesive performance), and very soft stalks are friction dominated (i.e. pillar adhesion is insignificant in comparison to the friction created by buckled stalks)^27^. The difference in shear adhesive performance between soft and stiff stalks is likely why gecko setae are not soft (i.e., avoid buckling, higher adhesive performance)^19,20^.

## Conclusion

The results of this study suggest that when geckos interact with environmental substrates that facilitate the formation of RH-induced water layers (i.e., hydrophilic substrates), capillary adhesion enhances adhesion (between 30–80% RH). When geckos interact with environmental substrates that do not facilitate the formation of RH-induced water layers (i.e., hydrophobic substrates), material softening enhances adhesion. Likewise, at low temperature, capillary adhesion influences gecko and GSA adhesive performance, but at high temperature material softening plays an important role. Taken together, these results show that capillary adhesion and material softening are important, highly contextual and non-mutually exclusive mechanisms geckos experience to enhance adhesion in particular circumstances. Although capillary adhesion appears to play a more dominate role in adhesive performance, few natural substrates are as hydrophilic as glass. Thus, functionally, it is likely geckos moving on hydrophobic leaves in high temperature and RH take advantage of slightly softer, more compliant setae that enhance adhesion at 32 °C, 70% RH, which are common climatic conditions in the tropics. Future work should explore adhesive performance of geckos native to drier and/or cooler climates to determine if this optimal peak is only found in tropical-dwelling geckos like the Tokay gecko (*Gekko gecko*) tested here. Likewise, the effect of setal surface chemistry is poorly understood, and may be an additional mechanism that geckos from multiple climates utilize to vary adhesive performance in complex environmental conditions.

## Materials and methods

### Experimental conditions

All gecko and GSA adhesion tests were conducted in a walk-in environmental chamber (Hotpack SP Scientific; Warminster, Pennsylvania, USA) with temperature and RH control (maintained at ± 2.0 °C and ± 5.0% RH of the set point). Two temperature and four RH set points (temperature: 12 °C and 32 °C; RH: 30%, 55%, 70%, and 80%) were used to create eight different experimental set point combinations, matching previous studies^34,47^. Experimental substrates and GSA samples were exposed to temperature and RH set points for 30 min prior to testing. Live geckos were acclimated for one hour to temperature and RH set points.

### Experimental substrates

Geckos and GSAs were tested on hydrophilic glass and hydrophobic octadecyltrichlorosilane-self assembled monolayer (OTS-SAM) coated glass (advancing contact angle = 100.1° ± 2.40 (s.d.), receding contact angle = 79.8° ± 1.58 (s.d.) for deionized water; s.d. = standard deviation). The OTS-SAM coated glass was made using a glass panel or a glass block (for GSA experiment) that was washed multiple times with deionized water and isopropyl alcohol. The glass was dried with nitrogen gas between each rinse. After preliminary rinses, the glass was soaked in a base bath for 3 h, rinsed with deionized water, and dried with nitrogen gas. The glass was then fully immersed in the OTS solution (1 mM of OTS in toluene) for 30 min. The container holding the solution and immersed glass was sealed to minimize atmospheric contact. After removing the substrate, a series of consecutive rinses were completed with toluene, acetone, chloroform, and isopropyl alcohol. Nitrogen gas was used to dry the substrate between each rinse. Finally, the substrates were left in a vacuum oven (ca. 150 °C) overnight to anneal. This process is described in more detail elsewhere (see SI Section 1 in^50^).

### Live animal adhesion experiments

Seven adult Tokay geckos (*G. gecko*) (body mass = 82.1 ± 17.35 g s.e.m.; s.e.m. = standard error mean) were obtained from California Zoological Supply and housed in the laboratory. Detailed husbandry and experimentation procedure has been reported previously^34^. Briefly, geckos were induced to securely position each foot on hydrophilic glass (56 × 33 cm panel) or hydrophobic OTS-SAM coated glass (25 × 15 cm panel) so that each toe pad was in full adhesive contact. We measured maximum shear adhesion, defined as the maximum shear adhesive force (N) a gecko can withstand before sliding. To measure maximum shear adhesion, geckos were harnessed around the pelvis with a thin flexible harness attached to a force sensor (Nidec Shimpo FGV-XY 100 N force gauge; Itasca, Illinois, USA) supported by a custom-built motorized apparatus (see^34^ for an example schematic). Geckos were pulled parallel to the vertical substrate at a constant rate (1.8 mm s^−1^) until all four feet begin to slide. The point where all four feet slip was logged as maximum shear adhesion and the trial was concluded. Maximum shear adhesion was measured three consecutive times for all geckos, however only the maximum shear adhesive force of the three trials was used in data analysis. In some trials, shear adhesive force was high and resulted in detachment of setae from the animal. If this occurred, all subsequent trials were discontinued, and the highest shear adhesive force value was used for analysis. After each adhesion test the substrate was cleaned with ethanol, then deionized water, and dried using a Kimtech wipe. Geckos were tested at a single environmental set point (temperature, RH) on a single substrate (hydrophilic or hydrophobic) per day. Geckos were given a minimum of 48 h to rest between trials. All geckos were randomly tested on each of the two types of substrates (hydrophilic and hydrophobic) at all eight experimental set points. A total of 112 data points were collected for live gecko experiments. Experimental procedure and housing of live geckos was in accordance with IACUC-1874 and IACUC-1878 (issued by Villanova University Institutional Animal Care and Use Committee). All methods were carried out in accordance with relevant guidelines and regulations.

### GSA fabrication and characterization

Mushroom GSAs (70 µm tip diameter, 50 µm stalk diameter, 50 µm stalk spacing, 100 µm pitch distance, 60 µm stalk height) were fabricated using a combination of soft molding techniques described elsewhere^51^. Samples with three characteristic stalk modulus states were obtained: soft, medium, and stiff. The soft GSA microfiber stalks were made with Ecoflex-00-30 and SU-8 lithographic templates. The prepolymer and curing agent were mixed (weight ratio of 1A:1B), degassed, and cast onto the SU-8 mold. The samples were cured at room temperature (23 °C) for 4 h and then demolded. Modulus of the soft GSA stalk material was 0.093 ± 0.0047 (s.d.) MPa and the static contact angle of the flat, non-structured cured Ecoflex-00-30 was 103.3° ± 2.13 (s.d.; using DI water). The medium GSA microfiber stalks were made with a mixture of PDMS (polydimethylsiloxane, Sylgard 184; weight ratio of 10:1) and Ecoflex-00-30 (weight ratio of 1A:1B; 80% and 20% by weight respectively). The mixture was degassed and cast onto the SU-8 lithographic templates. The samples were cured at 65 °C for 24 h and then demolded. The medium GSA stalk material modulus was 0.83 ± 0.020 (s.d.) MPa and the static contact angle of the flat, non-structured cured PDMS-Ecoflex mixture was 105.1° ± 2.35 (s.d.; using DI water). Finally, the stiff GSA microfiber stalks were made with PDMS and SU-8 lithographic templates. The pre-polymer and curing agent were mixed (weight ratio of 10:1), degassed, and cast onto the SU-8 mold. The samples were cured in a vacuum oven at 90 °C for 1 h and then demolded. The modulus of the stiff GSA stalk material was 1.91 ± 0.140 (s.d.) MPa and the static contact angle of the flat, non-structured cured PDMS was 105.0° ± 0.47 (s.d.; using DI water).

Mushroom-tip fabrication was identical for all three GSA stalk moduli used in this experiment. Specifically, a thin, homogeneous layer of vinyl siloxane (VS; Kulzer, Flexitime Medium Flow) precursor solution was coated over a glass plate by a film applicator (25–30 μm thickness). After partial crosslinking of the VS layer for 30–45 s, the micropatterned fibrillar patch (described above) was manually inked onto the thin layer and placed on a perfluorinated silicon wafer. Within a few minutes the viscous VS was crosslinked, peeled-off, and mushroom-shaped microfibers were created. DI water static contact angle of flat, non-structured VS was 13.5° ± 2.97 (s.d.). Although stalk modulus differed among GSA samples, the mushroom tips of the pillars were all made of VS and stiffer (modulus = 2.4 ± 0.09 (s.d.) MPa) than the stalk modulus of all three sample types. This ensured that the surface chemistry and contact mechanics of the adhesive contact interface did not change among samples^52^, nor were there additional contact mechanic consequences from different modulus gradients along the tip and stalks (i.e. the tips were always stiffer than the stalks)^53,54^. Varying only stalk modulus explores the mechanical consequences of the material softening hypothesis at the scale of the setal stalk, rather than the contact interface.

GSA fabrication and target moduli were achieved in accordance with previous work^55^. All modulus characterizations were measured using ISO527-2-type5b standards^55,56^. Stress–strain measurements were conducted with an Instron 5942 universal tensile tester (Norwood, MA, USA) set at a velocity of 200 µm s^−1^. Mechanical characterization was also measured on materials aged 2 months in the laboratory to test for an effect of aging on PDMS 10:1, Ecoflex 00–30, and PDMS—Ecoflex 00-30 mixture. There was no difference in the modulus of samples measured 1 day and 2 months after fabrication (Fig. S2).

Sessile drop measurements using a Krüss DSA100S goniometer (Hamburg, Germany) were used to measure the static water contact angle of experimental and sample substrates. In these characterizations, a ca. 2 µL deionized water droplet was deposited on the surface using a flat end needle (Sterican, 0.40 × 25 mm, Blunt Gauge 27). The DI water droplet was kept on the surface for 60 s. During this time, the images and measurements of the droplet were taken at 10 frames per seconds (fps) using a side-mounted camera. The average of the static contact angles was measured and calculated by goniometer software. For each sample, at least 10 different measurements were collected. Mechanical and static contact angle characterizations were conducted at room temperature (ca. 23 °C).

### GSA adhesion experiments

Square soft, medium, and stiff GSA samples (14.4 ± 1.89 (s.e.m.) mm^2^) were cut with a fine scalpel and glued to a clean glass microscope slide using silicon-based glue (Sil-Poxy; Macungie, Pennsylvania, USA). Samples were visually inspected using a dissecting microscope to ensure glue did not cover any part of the sample. Samples were dried for at least 24 h. Adhesion tests were conducted on the custom-built motorized apparatus described above with a force sensor (Nidec Shimpo XY 5 N force gauge; Itasca, Illinois, USA). The sample slide was attached to the experimental platform using double sided tape. A 106 g, 8 × 5 cm glass block or OTS-SAM coated glass block was placed on top of the sample, connected to the force gauge with a nylon string, and pulled in the shear direction at a constant rate (1.8 mm s^−1^), similar to previous studies^27,47^. Only the maximum shear force (N) recorded over a single 3 cm shear slide was used for data analysis. Maximum shear force of GSAs was then divided by individual sample area (N/mm^2^), in accordance with the previous work^27,47^. After each trial, the experimental substrate blocks were cleaned with ethanol, then deionized water, and dried using a Kimtech wipe. We tested shear adhesion of GSAs on each of the two types of substrates (hydrophilic and hydrophobic) at all eight temperature and RH set points. Five samples were used for each of the three modulus values (i.e., 15 total samples). Each sample was tested 16 times which generated 240 data points. Samples were tested between 3 and 16 months post fabrication. Sample test order and treatment was randomized to account for potential effects of PDMS ageing^57^. However, due to experimental constraints, all GSA trials conducted at 12 °C and 30% RH were tested last (i.e., sample order but not treatment order was randomized).

### Statistical analysis

We used a generalized linear mixed model (GLMM) to test the effect of temperature, RH, and substrate wettability on live gecko adhesive performance. Individual gecko identification number was used as a random factor. Bartlett’s test for homogeneity of variance on all explanatory variables (Table S3) showed heterogeneity between the two substrate wettability levels (hydrophilic and hydrophobic; K^2^ = 7.351, *p* = 0.006073). Therefore, we included a variance structure (*varIndent*) that allows for differences in the variances of adhesion of the two substrates. Data were natural log transformed to normalize the residuals of the model (Shapiro–Wilk test: W = 0.9847, *p* = 0.2298).

We also used a GLMM to test the effect of temperature, RH, substrate wettability, and modulus on adhesive performance of GSAs. Individual GSA sample identification number was included as a random factor. Bartlett’s test for homogeneity of variance on all explanatory variables (Table S4) showed heterogeneity between the three modulus levels (soft, medium, and stiff; K^2^ = 28.94, *p* < 0.0001). Therefore, we included a variance structure (*varIndent*) that allows for differences in the variances of soft, medium, and stiff GSA adhesion. Adhesion data were natural log transformed to normalize the residuals of the model (Shapiro–Wilk test: W = 0.9930, *p* = 0.3130).

GLMM models and HSD Tukey pairwise comparisons were carried out using the R packages *nlme*^58^ and *emmeans* respectively^59^. Statistical analyses and graphs were executed in *R*^60^.

## Supplementary information

Supplementary Information 1.

Supplementary Information 2.

## Data Availability

All data generated and analyzed for this study are included in this published article and its Supplementary Material files.
