# Supplementary material for: The effect of substrate wettability and modulus on gecko and gecko-inspired synthetic adhesion in variable temperature and humidity
Source: Sci Rep. 2020 Nov 12;10:19748. doi: 10.1038/s41598-020-76484-6 (PMC7665207; doi:10.1038/s41598-020-76484-6)
Supplement: Supplementary file 1 — Supplementary Information 1. [file 41598_2020_76484_MOESM1_ESM.docx]

**The effect of substrate wettability and modulus on gecko and gecko-inspired synthetic adhesion in variable temperature and humidity**

Christopher T. Mitchell^1,†^, Cem Balda Dayan^2,†^, Dirk-M. Drotlef^2^, Metin Sitti^2^, and Alyssa Y. Stark^1,*^

^1^ Department of Biology, Villanova University, 800 E. Lancaster Ave., Villanova, Pennsylvania, USA 19085

^2^ Physical Intelligence Department, Max Planck Institute for Intelligent Systems, Stuttgart, Germany 70569

† Contributed equally

* Correspondence to: alyssa.stark@villanova.edu

**Supplementary Material**

**
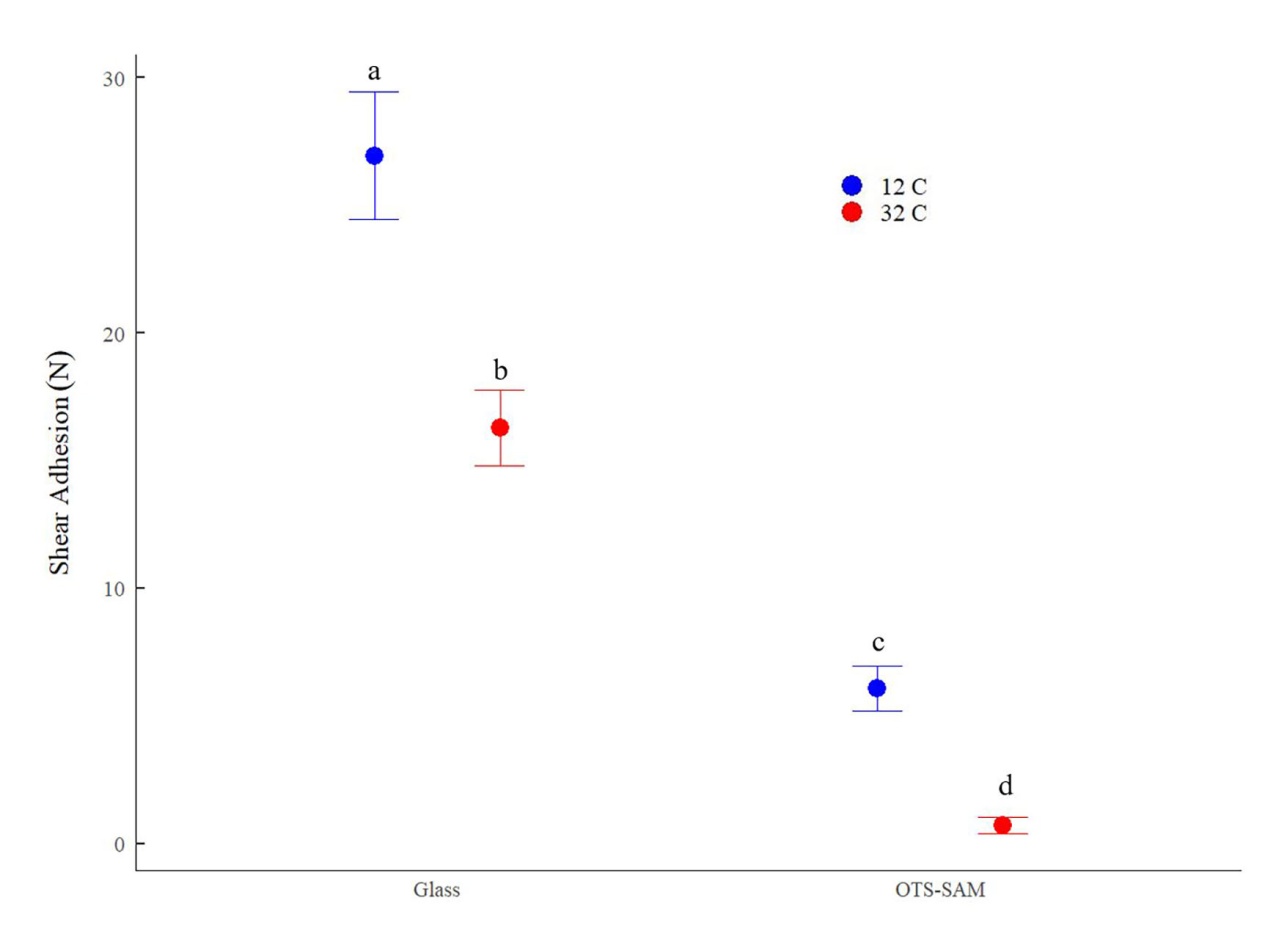
**

**Figure S1.** Shear adhesion (mean ± s.e.m.) of seven Tokay geckos (*Gekko gecko*) on hydrophilic (ca. 50° water contact angle) glass and hydrophobic (ca. 100° water contact angle) octadecyltrichlorosilane self-assembled monolayer (OTS-SAM) coated glass at two temperatures (12°C and 32°C). The means of treatment groups denoted with the same letter are not statistically different from one another according to Tukey post hoc pairwise statistical tests (see Table S1 for a detailed explanation of statistical analysis and the model output).

**
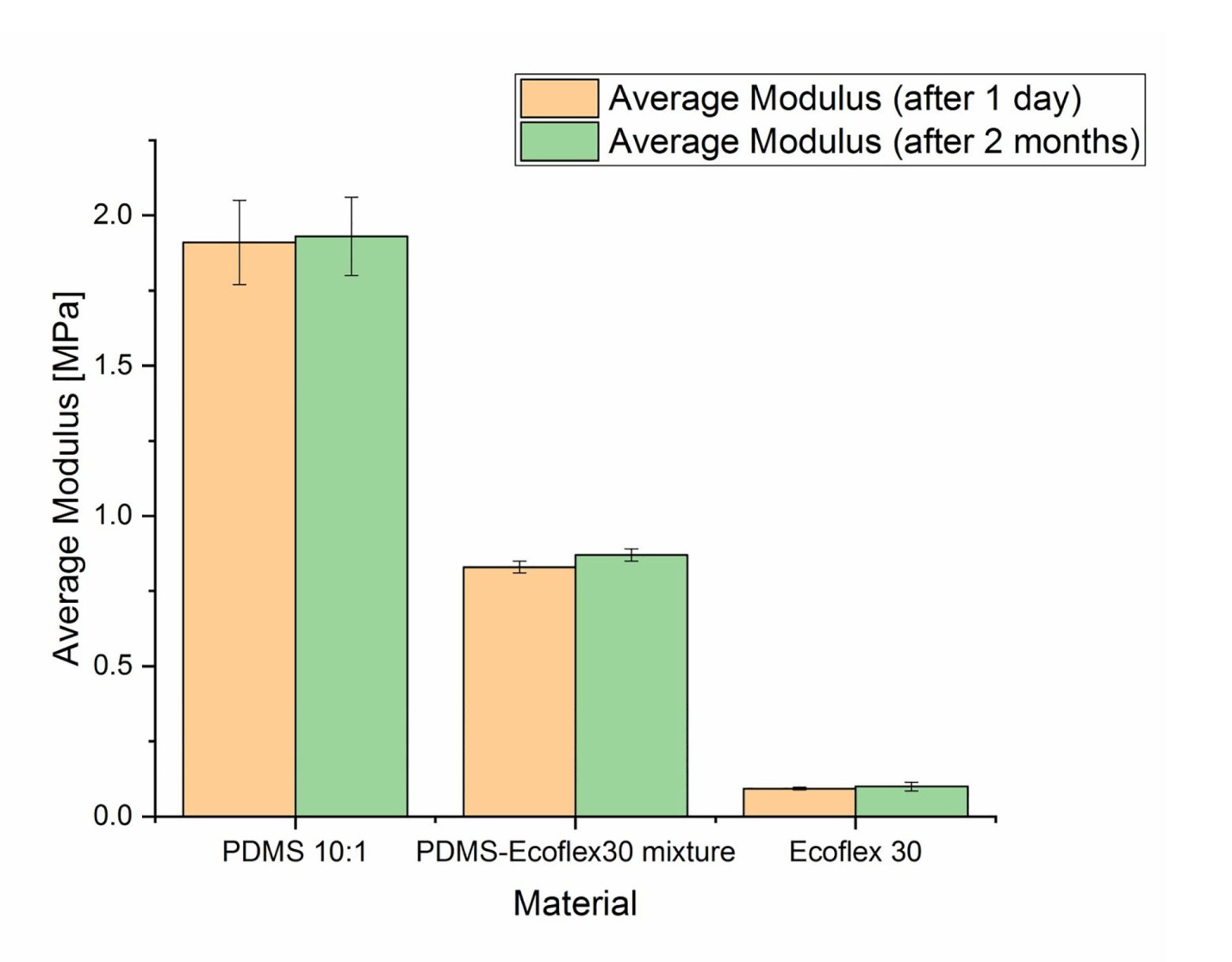
**

**Figure S2**. Aging test for modulus of PDMS 10:1, PDMS – Ecoflex 00-30 mixture (80 wt% and 20 wt%, respectively), and Ecoflex 00-30. There is no difference in Young’s modulus between samples aged 1 day and 2 months post-fabrication for PDMS 10:1, PDMS 10:1 and Ecoflex 00-30 mixture, and Ecoflex 00-30.

**Table S1.** GLMM model output of adhesive performance (natural log transformed) of seven Tokay geckos (*Gekko gecko*) tested in variable temperature (12°C, 32°C), humidity (30, 55, 70, 80% RH), and substrate wettability (hydrophobic, hydrophilic). All single factor and interaction test statistics are included. Statistically significant differences are in bold (i.e., p ≤ 0.05). A factor or interaction has at least one statistically significantly different mean if the corresponding p-value is p ≤ 0.05. This criteria means that there is at least a 95% probability that the given factor or interaction contributed to differences among means within the data.

| Model | Numerator degrees of freedom | Denominator degrees of freedom | F-value | p-value |
| --- | --- | --- | --- | --- |
| **Relative Humidity** | **3** | **90** | **26.5410** | **<0.0001** |
| **Temperature** | **1** | **90** | **74.3186** | **<0.0001** |
| **Substrate** | **1** | **90** | **476.3889** | **<0.0001** |
| **Relative Humidity: Temperature** | **3** | **90** | **10.1904** | **<0.0001** |
| **Relative Humidity: Substrate** | **3** | **90** | **5.0961** | **0.0026** |
| **Temperature: Substrate** | **1** | **90** | **32.0404** | **<0.0001** |
| Relative Humidity: Temperature: Substrate | 3 | 90 | 0.7167 | 0.5446 |

**Table S2.** GLMM model output of GSA adhesive performance (natural log transformed) tested in variable temperature (12°C, 32°C), humidity (30, 55, 70, 80% RH), substrate wettability (hydrophobic, hydrophilic), and constructed with different stalk moduli (soft, medium, stiff). All single factor and interaction test statistics are included. Statistically significant differences are in bold (i.e., p ≤ 0.05). A factor or interaction has at least one statistically significantly different mean if the corresponding p-value is p ≤ 0.05. This criteria means that there is at least a 95% probability that the given factor or interaction contributed to differences among means within the data.

| Model | Numerator degrees of freedom | Denominator degrees of freedom | F-value | p-value |
| --- | --- | --- | --- | --- |
| **Relative Humidity** | **3** | **180** | **15.032** | **<0.0001** |
| **Temperature** | **1** | **180** | **43.365** | **<0.0001** |
| **Substrate** | **1** | **180** | **23.565** | **<0.0001** |
| **Modulus** | **2** | **180** | **8.303** | **0.0054** |
| **Relative Humidity: Temperature** | **3** | **180** | **5.828** | **0.0008** |
| **Relative Humidity: Substrate** | **3** | **180** | **5.638** | **0.0010** |
| Temperature: Substrate | 1 | 180 | 3.094 | 0.0803 |
| Relative Humidity: Modulus | 6 | 180 | 1.237 | 0.2894 |
| Temperature: Modulus | 2 | 180 | 0.006 | 0.9936 |
| **Substrate: Modulus** | **2** | **180** | **24.610** | **<0.0001** |
| Relative Humidity: Temperature: Substrate | 3 | 180 | 0.700 | 0.5532 |
| Relative Humidity: Temperature: Modulus | 6 | 180 | 0.724 | 0.6304 |
| Relative Humidity: Substrate: Modulus | 6 | 180 | 0.456 | 0.8401 |
| Temperature: Substrate: Modulus | 2 | 180 | 0.089 | 0.9145 |
| Relative Humidity: Temperature: Substrate: Modulus | 6 | 180 | 0.538 | 0.7790 |

**Table S3.** Bartlett’s test for homogeneity of variance on all explanatory variables (natural log transformed) of seven Tokay geckos (*Gekko gecko*) tested in variable temperature (12°C, 32°C), humidity (30, 55, 70, 80% RH), and substrate wettability (hydrophobic, hydrophilic). Statistically significant tests are in bold (i.e., p ≤ 0.05). Significant tests indicate that the levels within the significant factor (i.e., substrate) have different variances, and therefore require additional consideration in a statistical model (i.e., a GLMM with *varIndent* function must be used).

| Explanatory variable | Bartlett’s K² | Degrees of freedom | p-value |
| --- | --- | --- | --- |
| Relative Humidity | 2.0078 | 3 | 0.5708 |
| Temperature | 3.0884 | 1 | 0.07885 |
| **Substrate** | **7.3509** | **1** | **0.006703** |

**Table S4.** Bartlett’s test for homogeneity of variance on all explanatory variables (natural log transformed) of GSA adhesive performance tested in variable temperature (12°C, 32°C), humidity (30, 55, 70, 80% RH), substrate wettability (hydrophobic, hydrophilic), and constructed with different stalk moduli (soft, medium, stiff). Statistically significant tests are in bold (i.e., p ≤ 0.05). Significant tests indicate that the levels within the significant factor (i.e. modulus) have different variances, and therefore require additional consideration in a statistical model (i.e., a GLMM with *varIndent* function must be used).

| Explanatory variable | Bartlett’s K² | Degrees of freedom | p-value |
| --- | --- | --- | --- |
| Relative Humidity | 1.5078 | 3 | 0.6805 |
| Temperature | 0.55574 | 1 | 0.456 |
| Substrate | 1.5664 | 1 | 0.2107 |
| **Modulus** | **28.936** | **2** | **<0.0001** |
